# A Triple-Filter NLOS Localization Algorithm Based on Fuzzy C-means for Wireless Sensor Networks

**DOI:** 10.3390/s19051215

**Published:** 2019-03-10

**Authors:** Long Cheng, Yifan Li, Yan Wang, Yangyang Bi, Liang Feng, Mingkun Xue

**Affiliations:** 1 Department of Computer and Communication Engineering, Northeastern University, Qinhuangdao 066004, China; 17732497527@163.com (Y.L.); wangyan_jgxy@neuq.edu.cn (Y.W.); zxam1099@yeah.net (L.F.); mingkunxue61@gmail.com (M.X.); 2 SANY GROUP CO., LTD., Changping, Beijing 102202, China; biyy@sany.com.cn

**Keywords:** wireless sensor network, non-line-of-sight, residual analysis, Kalman Filter, Unscented Kalman Filter, Fuzzy-C-Means

## Abstract

With the rapid development of communication technology in recent years, Wireless Sensor Network (WSN) has become a promising research project. WSN is widely applied in a number of fields such as military, environmental monitoring, space exploration and so on. The non-line-of-sight (NLOS) localization is one of the most essential techniques for WSN. However, the NLOS propagation of WSN is largely influenced by many factors. Hence, a triple filters mixed Kalman Filter (KF) and Unscented Kalman Filter (UKF) voting algorithm based on Fuzzy-C-Means (FCM) and residual analysis (TF-FCM) has been proposed to cope with this problem. Firstly, an NLOS identification algorithm based on residual analysis is used to identify NLOS errors. Then, an NLOS correction algorithm based on voting and NLOS errors classification algorithm based on FCM are used to process the NLOS measurements. Hard NLOS measurements and soft NLOS measurements are classified by FCM classification. Secondly, KF and UKF are applied to filter two categories of NLOS measurements. Thirdly, maximum likelihood localization (ML) is employed to estimate the position of mobile nodes. The simulation result confirms that the accuracy and robustness of TF-FCM are better than IMM, UKF and KF. Finally, an experiment is conducted to test and verify our algorithm which obtains higher localization accuracy.

## 1. Introduction

The most popular worldwide localization is the Global Positioning System (GPS) which is used to tackle outdoor positioning problems, but its accuracy cannot meet requirements in some areas especially in indoor environments. Therefore, Wireless Sensor Network (WSN) based on indoor localization has attracted attention in recent years.

As a research hotspot, WSN has been widely applied in military applications, environmental monitoring, medical care and space exploration. Moreover, the localization techniques based on a mobile node’s location has vital research significance [1]. Because of the influence of Non-Line-of-Sight (NLOS) propagation, and other factors such as insufficient position signal strength, electromagnetic interference noise and mobile beacon node, the accuracy of localization has become a critical research direction.

According to measuring the distance between nodes, localization algorithms are normally divided into two categories: range-based algorithms and range-free algorithms, and there are many conventional methods in range-based algorithms; for example, time difference of arrival (TDoA), time of arrival (ToA), received signal strength (RSS), direction-of-arrival (DoA) and space-division multiple-access (SDMA) exploiting rich multi-path environment (i.e., NLOS) [2]. There are two categories of nodes in WSN: beacon nodes and mobile nodes. The coordinate information of beacon nodes can be acquired, while the position of mobile nodes is unknown. The position of a mobile node can be estimated by using the algorithms mentioned above.

The biggest challenge of indoor localization is NLOS propagation. The obstacles between beacon nodes and mobile nodes are the major factors generating NLOS errors, mostly because the obstacles block or reflect the spread of the signal. Therefore, in range-based indoor localization, the mitigation of NLOS errors is profoundly critical to improve the indoor localization accuracy. Many strategies have been proposed in order to mitigate NLOS effects [3,4,5,6,7,8,9,10,11,12,13].

In order to tackle the fluctuation problem of range information, many researchers have proposed various filtering algorithms: Average Filtering algorithm (AF), Gauss Filter (GF), Kalman Filter (KF), Extended Kalman Filter (EKF), Unscented Kalman Filter (UKF), Particle Filtering (PF) and so on. This paper proposes a triple filter algorithm based on KF and UKF. Most papers divide measurements into two categories: LOS and NLOS. Actually, the measurements with NLOS errors can be classified more meticulously so as to better cope with the NLOS problem. Considering that KF has limitations in dealing with nonlinear noise, UKF is used to cope with non-linear noise. The main contributions of this paper are as follows:

(1) A residual analysis based on the NLOS identification algorithm is proposed to identify NLOS errors. A method of mixed UKF and KF is proposed to filter out measurements noise, and no prior information is required in this mixed algorithm, which can better mitigate NLOS errors.
(2) The NLOS correction algorithm based on voting is proposed to restrict NLOS errors and correct the NLOS measurements, which are used to preprocess the NLOS measurements that need to be classified.
(3) NLOS measurements are classified into two categories: hard NLOS measurements and soft ones by Fuzzy-C-Means (FCM), which can be extended into more filters to tackle complicated NLOS errors, and it is flexible and robust as well.
(4) The algorithm is applied to different NLOS conditions, such as Gaussian Distribution, Exponential Distribution, and Uniform Distribution, which make our algorithm robust to a complex environment. An experiment has been conducted, and the result shows that our algorithm has better performance compared with KF, IMM and UKF.

This paper is organized as follows. Firstly, the symbol description will be given in Section 2. The related works are described in Section 3. Then, some descriptions about problem statement and a brief introduction of UKF and KF are described in Section 4. Next, our algorithm is presented in detail in Section 5, and Section 6 will show the simulation and experiment results of contrast in the midst of our algorithm and other methods. Finally, the conclusions are made in Section 7. 

## 2. Symbol Description

Key notions are shown in Table 1.

## 3. Related Works

With the extensive research on WSN, many researchers have proposed algorithms to deal with the NLOS condition. Among most methods, LOS and NLOS measurements are classified to mitigate the influence of NLOS errors and improve the accuracy of positioning. These methods are mainly range-based algorithms, which need point to point distance or angle information to estimate the location. In [3], Liao proposed the classic Interacting Multiple Model (IMM), which is regarded as the classical algorithm to tackle NLOS problems. The significant contribution of [3] is a two-state Markov switching model, which is used to measure the switching probability of NLOS and LOS measurements. In addition, the combination probability of IMM is updated by Markov switching probability. However, the NLOS identification method of IMM is fuzzy, which is not accurate enough to distinguish between NLOS and LOS errors. Furthermore, more NLOS parameter information and prior information is needed when filtering, which represents a lack of strong robustness. In [4], Huerta proposed a joint PF and an UKF algorithm (RBPF). In this paper, a Bayesian model is used to fully exploit the LOS and NLOS measurements. PF and UKF are used to estimate the trajectory and kinetic variable (position and speed) of a mobile node. Although the experimental result shows great performance, the complex and comprehensive simulation scenario is needed to illustrate the robustness of the algorithm. Tomic proposed a target localization algorithm utilizing integrated measurements; namely, RSS and AoA in [5]. Through convex relaxations and derivation of convex problems, global minima and solutions can be obtained. The most important contribution of his work is combined RSS and AoA, which produces a high-precision and -accuracy of localization, but the device complexity is high, and the identification of NLOS and LOS needs to be improved. 

In addition, there are several studies based on ToA [6,7,8]. Tomic proposed a bisection-based approach for exact target localization in NLOS environments [6]. In this paper, he utilized the maximum likelihood (ML) estimator and converted the localization problem into a generalized trust region sub-problem (GTRS) framework. It has the same performance as existing methods, but the computational complexity is better. The great advantage of the proposed algorithm is its linear computational complexity. Nevertheless, its accuracy and precision need to be further improved. Pak proposed distributed hybrid particle/FIR filtering (DHPFF) for mitigating NLOS effects in TOA-based localization using wireless sensor networks in [7]. In this paper, the hybrid particle finite impulse response filter (HPFF) was adopted in order to cope with the cases that NLOS noises generate. The advantage of this algorithm is that it can achieve self-recovery by detecting failures and resetting the algorithm. However, the computational complexity of DHPFF is too high to be applied to computers with poor computational power. Liu proposed a closed form localization method using AoA and ToA measurements based on WSN in NLOS environments in [8]. This paper used a two-step weighted least square (WLS) estimator by means of the first-order error analysis. However, an experiment is needed, because the Gaussian distribution of simulation cannot cover all the complex situation in the real world.

NLOS identification is profoundly significant in most range-based algorithms. Hence, some methods have been proposed to address this issue better [9,10,11,12,13]. Hua proposed an NLOS identification and positioning algorithm based on a localization residual in WSN in [9]. A two-step algorithm to mitigate NLOS errors is proposed, including residual analysis addressing the positioning difference and a threshold hypothesis test. The simulation shows a good performance, but the algorithm needs an experiment based on a real scenario, and the hypothesis based on threshold needs much reliance on people’s subjective experience. Yang proposed NLOS identification for UWB localization based on import vector machine (IVM) in [10]. In his paper, a novel NLOS identification algorithm with feature selection strategy has been put forward so as to ameliorate the classification accuracy. Besides, a localization algorithm based on IVM with high accuracy and low complexity is also a highlight, which produces higher positioning accuracy than Support Vector Machine (SVM) and Relevance Vector Machine (RVM), but the algorithm needs plenty of training data, and will perform poorly with few training sets. Lei come up with a robust cooperative indoor localization based on reliability evaluation in [11]. The algorithm combines the advantages of compressive sensing (CS) in the LOS condition and the PF in the NLOS condition, and a reliability evaluation is also proposed to evaluate the estimation results of CS and PF. However, the NLOS identification of this method is based on reliability evaluation, which is not accurate to distinguish the NLOS and LOS measurements. Aghaie presented localization of WSN nodes based on NLOS identification using AoAs statistical information in [12]. The main idea of this paper is the identification of NLOS nodes based on the statistical model of the measurement and the NLOS errors where they utilized an NP method to judge a threshold. Yu put forward a mean shift-based mobile localization method in mixed LOS/NLOS environments for a wireless sensor network in [13]. A novel mobile localization method is presented in order to overcome the effects of NLOS errors by utilizing the mean shift-based Kalman filter, but the NLOS identification is based on the hypotheses and alternatives methods. These methods need prior thresholds.

Apart from the Maximum Likelihood (ML) based on the parameter estimation methods mentioned above, there are also other popular approaches in the family of ML-based parameter estimation. The iterative Space-Alternative Generalized Expectation Maximization Algorithm (SAGE) introduces some hidden data to simplify the ML estimation [14]. A soft-input soft-output (SISO) version improves robustness, which is applied to joint successive interference cancellation and channel estimation [15]. The iterative method is called multiple-input multiple-output space division multiple access (MIMO-SDMA), which approaches the maximum likelihood estimate with fast convergence and low complexity [16].

## 4. Problem Statement

This section is made up of three parts: signal model and brief introductions of UKF and FCM. In Section 4.1, some necessary assumptions and important definitions about NLOS are proposed to better describe the algorithm. In Section 4.2 and Section 4.3, a brief presentation about the methods is given, including FCM and UKF. In Section 4.2, a brief description of UKF is made, which is used to cope with NLOS errors. In Section 4.3, an introduction about FCM is used to classify NLOS measurements into two categories: hard NLOS measurements and soft ones.

### 4.1. Signal Model

In this section, some technical preparations are introduced to describe the algorithm. N beacon nodes are randomly distributed in field with the coordinates given by $\left(x_{i},y_{i}\right)$, i=1,…,N. The coordinate of unknown node at the time k is (x(k),y(k)), $t=t_{0},\ldots ,t_{n}.$ Hence, the true distance $d_{i}(k)$ between the i-th unknown node and the beacon node at time k [17,18,19] can be expressed as:

(1) $$d_{i}(k)=\sqrt{{\left(x_{i}-x(k)\right)}^{2}+{\left(y_{i}-y(k)\right)}^{2}}$$

Due to the existence of measurement errors, the noise of actual measurement distance ${\hat{d}}_{i}^{LOS}(k)$ under LOS condition can be approximated by Gaussian white noise. So the LOS measurement distance can be expressed as follows:

(2) $${\hat{d}}_{i}^{LOS}(k)=d_{i}(k)+n_{LOS}$$

where $n_{LOS}^{}$ is the measurement noise obeyed Gaussian Distribution with zero mean and $\sigma_{LOS}^{2}$, i.e., $N(0,\sigma_{LOS}^{2}).$ The probability density function of $n_{LOS}$ can be expressed as:

(3) $$f(n_{LOS})=\frac{1}{\sqrt{2\pi \sigma_{LOS}^{2}}}\exp(-\frac{n_{LOS}^{2}}{2\sigma_{LOS}^{2}})$$

In actual scenario, there are obstacles between beacon nodes and mobile node, which result in the propagation distance is longer than true distance. Thus, some NLOS noise $n_{NLOS}$ can be added to measurement distance ${\hat{d}}_{i}^{NLOS}(k)$:

(4) $${\hat{d}}_{i}^{NLOS}(k)=d_{i}(k)+n_{LOS}+n_{NLOS}$$

Moreover, the NLOS error $n_{NLOS}$ is usually regarded as positive, which obey Gaussian, Uniform, or Exponential Distribution. The probability density function of $n_{NLOS}^{norm}$ obeyed Gaussian Distribution ($n_{NLOS}^{norm}∼N(\mu_{NLOS},\sigma_{NLOS}^{2})$) can be described as follows:

(5) $$f(n_{NLOS}^{norm})=\frac{1}{\sqrt{2\pi \sigma_{NLOS}^{2}}}\exp(-\frac{{\left(n_{NLOS}^{norm}-\mu_{NLOS}\right)}^{2}}{2\sigma_{NLOS}^{2}})$$

And the probability density function of $n_{NLOS}^{unif}$ which obeys to Uniform Distribution ($n_{NLOS}^{unif}∼U(u_{\min},u_{\max})$) is given by:

(6) $$f(n_{NLOS}^{unif})=\{\begin{array}{cc} \frac{1}{u_{\max}-u_{\min}}, & u_{\min}\leq n_{NLOS}^{unif}\leq u_{\max}\\ 0, & else \end{array}$$

The probability density function of $n_{NLOS}^{\exp}$ which obeys to Exponential Distribution ($n_{NLOS}^{\exp}∼E(\lambda )$) is given by:

(7) $$f(n_{NLOS}^{\exp})=\{\;\begin{array}{cc} \frac{1}{\lambda }e^{-\frac{n_{NLOS}^{\exp}}{\lambda }}, & n_{NLOS}^{\exp}\geq 0\\ 0, & n_{NLOS}^{\exp}<0 \end{array}$$

### 4.2. A Brief Introduction to UKF

In our algorithm, UKF is utilized to tackle NLOS measurements including hard NLOS measurements and soft ones. In this section, the advantages of UKF are firstly introduced. Then, an important technique UT transform that is used in UKF will be discussed to better introduce UKF. Finally, the brief implementation steps of UKF will be given.

UKF is an algorithm that uses sampling points to estimate nonlinear distribution and its main frame is based on KF. KF is the most commonly used filter in WSN localization, but KF is proposed to solve problems based on a linear system that the noise is assumed as Gaussian white noise, and it is ineffective when filtering non-linear noise. After that, researchers proposed an algorithm to linearize nonlinear systems, which is known as Extended Kalman Filter (EKF), but the linear processing of EKF causes great calculation errors, even causing the filtering results not to converge. Moreover, EKF needs to calculate Jacobi matrix increasing the amount of calculation. Later, Simon J. Julier introduced Unscented Transformation (UT) to the analysis of KF, which obtained a higher precision and a lower algorithm complexity than EKF.

UT is the most important part of UKF that is different from EKF. UT is a conversion when calculating statistical characteristics of random variables in a nonlinear transformation. UT calculates posterior statistical properties of random vectors by means of weighted statistical linear regression. The selection of the sampling strategy in the UT transform has a vital impact on the estimation results. The sampling strategy of UT includes symmetric sampling, simplex sampling, third-order moment skew sampling, Gaussian distribution fourth-order moment symmetric sampling, etc. The difference between the sigma points produced by the various sampling strategies is the distance and the weight to the center point.

UKF is based on UT, abandoning the traditional method and using KF as its main frame. For one-step prediction equations, UKF uses UT to handle the nonlinear transfer of mean and covariance. More importantly, UKF approximates the probability density distribution of nonlinear function, and uses a series of determined samples to approach the priori probability density of the state, rather than approximating the nonlinear function. UKF does not linearize to ignore high order terms, therefore, the nonlinear distribution statistic has higher calculation accuracy [20,21,22,23].

Above all, UKF is an iterative estimation algorithm selecting sigma points according to feature distribution of sampling points. These sigma points iterate through a state equation and become the prediction points. The results are constantly corrected by Kalman gain and the errors between the true value and the measurements. Finally, the optimal estimated value can be obtained [24].

### 4.3. A Brief Introduction of FCM

FCM is used to classify the NLOS measurements into two categories: hard NLOS measurements and soft ones. In this section, firstly, the main feature of FCM will be discussed. Then, some basic concepts used in FCM will be explained, namely, fuzzy sets and membership function. Finally, the advantages of FCM are given to illustrate that it is appropriate to use FCM in our algorithm.

FCM is a commonly used clustering algorithm. According to a similarity criterion, a set of sample datasets without class identification is divided into several subsets. The Fuzzy C-Means (FCM) algorithm is a partition-based clustering algorithm. Its main idea is to maximize the similarity among objects divided into the same cluster, and minimize the similarity among different clusters. FCM is the improvement of the ordinary C-Means algorithm. The traditional C-Means is Crisp Partition such as K-Means, while the FCM is based on a fuzzy and flexible division according to the membership of each element. FCM clustering is an unsupervised method of gradual optimization based on iterative thinking. This method is to use the least squares principle to iteratively optimize the objective function so as to realize the fuzzy division of the dataset. On one hand, its local convergence property is very good. On the other hand, the algorithm is also effective for high dimensional characteristic space [25].

Fuzzy sets and membership function are the most important concepts of FCM. The membership function indicates the extent to which an object x belongs to the set A, which is usually expressed as $\mu_{A}(x)$. Its independent variable range is all objects that may belong to set A (which means all points in the space where set A is located), and the value range is [0,1], that is $0\leq \mu_{A}(x)\leq 1$. Thus, $\mu_{A}(x)=1$ means x is fully affiliated to set A, which is the equivalent of the traditional set concept x∈A. A membership function defined in space X can be given as a fuzzy set A, or can be called as a fuzzy subset defined in space X. With the concept of fuzzy sets, it is not hard for an element that belongs to a fuzzy set. In the clustering problem, clusters generated by clusters can be regarded as fuzzy sets. As a result, the membership value of each sample point $x_{i}$ is in accordance with the following relationship: $0\leq \mu_{A}(x_{i})\leq 1$.

FCM divides n-dimensional vectors $x_{i}(i=1,\ldots ,n)$ into c fuzzy groups, and finds the cluster center of each group, so that the value function (or objective function) of the non-similarity index is minimized. The advantages of FCM mainly include the following aspects:

- The idea of FCM can objectively reflect the authenticity of things and comply with the characteristics of human cognition of things.
- After the given initial parameters, FCM does not need manual intervention in clustering, which is an unsupervised process. It can complete clustering only by dataset, which is more suitable for automatic processing.
- FCM is fuzzy, especially suitable for the problems of uncertainty and ambiguity.

## 5. Proposed Method

**Figure 1:**
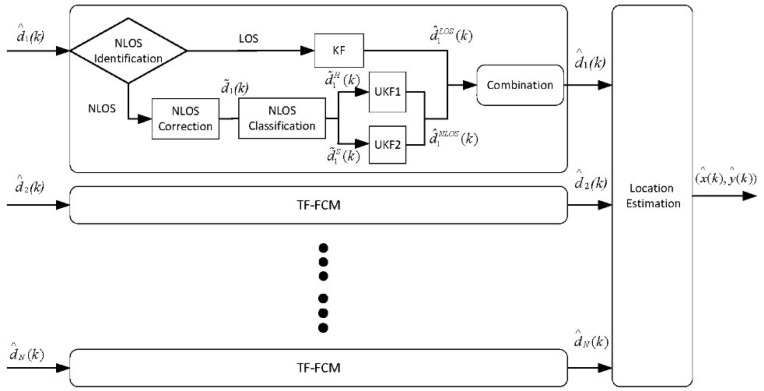
The flowchart for the proposed algorithm.

The flow chart of our algorithm is shown as Figure 1. The input of TF-FCM is ${\hat{d}}_{i}(k)$ (i=1,2,3,…,N). ${\hat{d}}_{i}(k)$ indicates the measurement at time k, and N indicates the number of the beacon nodes. The output of TF-FCM is the prediction coordinate ${\hat{P}}_{i}(k)(x(k),y(k))$. Firstly, the NLOS identification algorithm based on residual analysis is proposed to identify the propagation conditions. Then, if the propagation is NLOS condition, KF is presented to cope with the measurements. For the NLOS situation, NLOS correction algorithm based on voting is proposed to deal with it in advance, and the processed measurements after voting will be classified by NLOS classification based on FCM. Hard NLOS measurements and soft ones will be classified after FCM. Hard NLOS measurements will be processed by UKF1, and the soft NLOS measurements will be processed by UKF2. The NLOS measurements after filtering can be obtained by calculating arithmetic average. Finally, the LOS and NLOS processed measurements will be combined by weights. The processed measurements are used to calculate coordinates of a mobile node by maximum likelihood (ML) localization.

### 5.1. NLOS Identification Based on Residual Analysis

In case that the NLOS errors data is unknown, this paper assumes that there are N(N≥3) measured distances from N beacon nodes. If each group has no more than three distances, $N_{C}$ different combinations can be calculated to group N measurements:

(8) $$N_{C}=\sum_{i=3}^{N}C_{N}^{i}$$

Then, the index of every combination is defined as $\left\{S_{k}|k=1,2,\ldots ,N_{C}\right\}$. After estimating each node’s coordinate by location estimation, the estimated position ${\hat{X}}_{i}$ of every combination $S_{k}$ and corresponding residual Res can be obtained. The residual of $S_{k}$ is given by:

(9) $$Res({\hat{X}}_{k},S_{k})=\frac{\sum_{i\in S_{k}}^{}{\left(d_{i}-\left\|{\hat{X}}_{k}-P_{i}\right\|\right)}^{2}}{N_{C}}$$

And the average residual can be defined as:

(10) $$\bar{Res}({\hat{X}}_{k},S_{k})=\frac{\sum_{k=1}^{N_{C}}Res(X_{k},S_{k})}{N_{C}}$$

Average residual $\bar{Res}({\hat{X}}_{k},S_{k})$ is utilized to compare with the process noise $\sigma_{i}$ to identify the propagation status of the measurements.

(11) $${\hat{s}}_{i}(k)=\{\begin{array}{cc} 1, & \bar{Res}({\hat{X}}_{k},S_{k})\leq \sigma_{i}\\ 2, & \bar{Res}({\hat{X}}_{k},S_{k})>\sigma_{i} \end{array}$$

where the ${\hat{s}}_{i}$ indicates the state (1:LOS, 2:NLOS) of the measurements.

The description of residual analysis is as follows (Algorithm 1):

**Algorithm 1**. Description of Residual AnalysisInput: ${\hat{d}}_{k}^{i}$ Output: ${\hat{s}}_{i}$ begin for *i* = 1 : *Size of*
$S_{k}$ do  $Res=Res+{\left({\hat{d}}_{i}(k)-\left\|{\hat{X}}_{k}-P_{i}\right\|\right)}^{2}/size(S_{k})$
end for for *i =* 1: $N_{C}$ do $\bar{Res}=\bar{Res}+Res_{k}$ end for $\bar{Res}=\frac{\bar{Res}}{N_{C}}$ if $\bar{Res}\geq \sigma_{i}$ then  ${\hat{s}}_{i}=2$
else  ${\hat{s}}_{i}=1$ end if end

### 5.2. NLOS Correction Algorithm Based on Voting

If measurement ${\hat{d}}_{i}$ is identified as NLOS, which means ${\hat{d}}_{i}$ contains many NLOS errors, a voting algorithm is used to mitigate the influence NLOS errors. The voting matrix is constructed to provide the initial estimated location to obtain the correction distance. To begin with, assume the area of the field is M×M, and it can be divided into X×X cells which depend on the estimation accuracy and computational complexity of the algorithm [22], and the location of each grid can be expressed as C(m,n), for m,n=1,2,…,X. The resolution of each grid is defined as $w=\frac{M}{X}$. For instance, if the field is 100×100 and X=100, the resolution of cells is equal to: $\frac{100}{100}=1$.

The algorithm consists of the following steps.

**Step 1**: An X×X voting matrix V is constructed to estimate the distance, and the elements representing voting results can be expressed by:

(12) $$V(m,n)=\sum_{i=1}^{N}b_{i}(m,n),\;for\;m,n=1,2,\ldots ,X$$

where $b_{i}(m,n)$ indicates the number of notes increased at each grid, which can be defined as:

(13) $$b_{i}(m,n)=\{\begin{array}{cc} 1, & {\hat{d}}_{i}-3\varepsilon \leq d_{imn}\leq {\hat{d}}_{i}+3\varepsilon \\ 0, & otherwise \end{array}$$

where $d_{imn}$ represents the Euclidean distance between i-th beacon node and C(m,n). ${\hat{d}}_{i}$ is the measurement distance of the i-th beacon node. The value of ε can be adjusted to make estimation more accurate, and here $\varepsilon =\sigma_{LOS}$.

**Step 2**: The largest value in the voting matrix can be obtained, which is marked as $V(m^{*},n^{*})$, and $V(m^{*},n^{*})\geq V(m,n),\;for\;m,n=1,2,\ldots ,X$. The estimated location of mobile node is $C^{*}=[C_{1}^{*},C_{2}^{*},\ldots ,C_{v}^{*}]$, where $C^{*}$ is the initial results set and v represents the number of elements in $C^{*}$, and the estimated location of the mobile node is given by:

(14) $${\bar{C}}^{*}=\sum_{i=1}^{v}\frac{C_{i}^{*}}{v}$$

**Figure 2:**
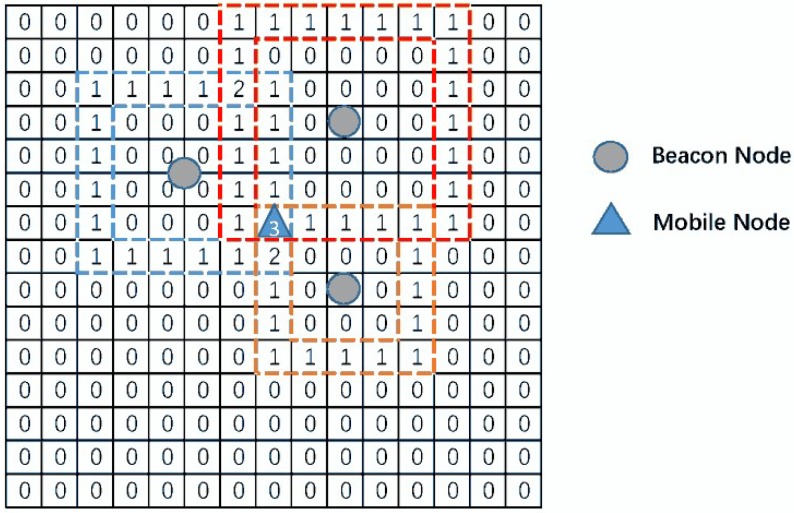
The Description of Voting.

The brief description of **Step 2** is illustrated as Figure 2. The vote of each grid will increase if the measurement area of beacon nodes is overlapped.

**Step 3**: The corrected distance ${\tilde{d}}_{i}(k)$ of the i-th beacon node can be updated as:

(15) $${\tilde{d}}_{i}(k)=\left\|{\bar{C}}^{*}-P_{i}\right\|$$

where $P_{i}=[x_{i},y_{i}]$ represents the location of the i-th beacon node.

Based on the aforementioned descriptions, the pseudo code of Voting can be summarized as Algorithm 2: 

**Algorithm 2**. Description of NLOS Correction Algorithm based on VotingInput: ${\hat{d}}_{i}(k)$
Output: ${\tilde{d}}_{i}(k)$ begin  V=0
for *i* = 1 : *N* do  for *m* = 1 : *W* do    for *n* = 1 : *W* do      $d_{imn}=\left\|C(m,n)-P_{i}\right\|$
    end for  end for  $V(m,n)=V(m,n)+b_{i}(m,n)$ end for $C^{*}=[m^{*},n^{*}]=max(V)$ $v=size\;(C^{*})$ ${\bar{C}}^{*}=\sum_{i=1}^{v}\frac{C_{i}^{*}}{v}$ for *i* = 1:*N* do  ${\tilde{d}}_{i}(k)=\left\|{\bar{C}}^{*}-P_{i}\right\|$ end for end

### 5.3. NLOS Errors Classification Based on FCM

Because the real distribution of NLOS is complex, it is impractical to know the parameters of NLOS errors. In order to tackle this problem, FCM is used to approximate the arbitrary probability density by adjusting its parameters.

Let ${\tilde{d}}_{i}(k)$ denote the range from i-th beacon node at time k after voting. M measurements from the i-th beacon node can be obtained over a time slot [k−δ,k+δ]. In order to make our statement simpler, time interval k is introduced to indicate the time slot, for example, ${\tilde{d}}^{i}(k)=[{\tilde{d}}^{1}(k),{\tilde{d}}^{2}(k),\ldots ,{\tilde{d}}^{M}(k)]$. Let c denote the number of cluster centers (1<c<M). Here, we set c=3. Let $v=\{v_{1},v_{2},\ldots ,v_{c}\}$ indicate cluster centers set. Let $\mu_{ij}$ indicate the membership between the i-th distance ${\tilde{d}}_{i}(k)$ at time k and the j-th cluster center $v_{j}$, and the membership matrix is given by $U=[\mu_{ij}]$ whose size is c×M. $d_{ij}$ indicates the Euclidean distance between ${\tilde{d}}^{j}(k)$ and $v_{i}$: $d_{ij}=\left\|v_{i}-d^{j}(k)\right\|$, and FCM can be expressed as a mathematical planning problem:

(16) $$min\;J(U,c)=\sum_{i=1}^{c}\sum_{j=1}^{M}{\left(\mu_{ij}\right)}^{m}d_{ij}^{2}$$

where *m* represents the fuzzy weights factor and $\mu_{ij}$ meets the following formula:

(17) $$\{\begin{array}{cc} \sum_{i=1}^{M}\mu_{ij}=1, & i=1,2,\ldots ,M\\ \sum_{i=1}^{c}\mu_{ij}<c, & 1\leq i\leq c\\ \mu_{ij}\in [0,1] & \end{array}$$

Cluster center $v_{i}$ can be derived as follows to calculate the minimum value of the objective function based on the Lagrange multiplier method.

(18) $$v_{k}=\frac{\sum_{i=1}^{M}\mu_{ij}^{m}{\hat{d}}^{i}(k)}{\sum_{i=1}^{M}\mu_{ij}^{m}},\;1\leq k\leq c$$

The sorted cluster center $v=\{v_{1},v_{2},\ldots ,v_{c}\}$ can be obtained. Since the NLOS error is of a positive bias and the largest value contains many NLOS errors, so the final result is given by:

(19) $$v=\{v_{1},v_{2},\ldots ,v_{c-1}\}$$

Hence, the classified distance can be described as follows, and the measurements are divided into two categories: hard NLOS measurements and soft ones.

(20) $$\{\begin{array}{ccc} {\hat{d}}_{i}^{h}(k)=v_{1} & {\hat{d}}_{i}^{s}(k)=v_{2}, & if\;v_{1}>v_{2}\\ {\hat{d}}_{i}^{h}(k)=v_{2} & {\hat{d}}_{i}^{s}(k)=v_{1}, & else \end{array}$$

Based on the aforementioned descriptions, the NLOS error classification algorithm based on FCM can be summarized as the pseudo code shown in Algorithm 3:

**Algorithm 3**. Description of NLOS Errors Classification Algorithm based on FCMInput: ${\tilde{d}}_{i}(k)$
Output: ${\tilde{d}}_{i}^{h}(k){\tilde{d}}_{i}^{s}(k)$ begin while 1 do  for *i* = 1 : *c* do    for *j* = 1 : *M* do      $d_{ij}=\left\|v_{i}-d^{j}(k)\right\|$
      $J(U,c)=J(U,c)+\mu_{ij}^{m}d_{ij}^{2}$
    end for  end for  if $\left\|J(U,c)-J_{0}(U,c)\right\|<\delta$ then    $v_{k}=\sum_{i=1}^{M}\mu_{ij}^{m}{\hat{d}}^{i}(k)/\sum_{i=1}^{M}\mu_{ij}^{m}$    $sort\{v_{1},\ldots ,v_{3}\}$   if $v_{1}>v_{2}$ then     ${\tilde{d}}_{i}^{h}(k)=v_{1}$     ${\tilde{d}}_{i}^{s}(k)=v_{2}$    else     ${\tilde{d}}_{i}^{h}(k)=v_{2}$     ${\tilde{d}}_{i}^{s}(k)=v_{1}$    end if     break  end if end while

### 5.4. Kalman Filter

For LOS, KF is used to process LOS measurements. To explain the state more precisely, a state vector of the i-th beacon node is defined as:

(21) $$X_{i}(k)=[{\hat{d}}_{i}(k),{\dot{d}}_{i}(k)]$$

where ${\hat{d}}_{i}(k)$ indicates the distance between the i-th beacon node and the mobile node and ${\dot{d}}_{i}(k)$ indicates the velocity that ${\hat{d}}_{i}(k)$ varies.

The state equation of the i-th beacon node under NLOS can be denoted as follows:

(22) $$X_{i}(k)=FX_{i}(k-1)+Cw_{i}(k)$$

where $F=\left[\begin{array}{cc} 1 & \Delta t\\ 0 & 1 \end{array}\right],\;C=\left[\begin{array}{c} \Delta t^{2}/2\\ \Delta t \end{array}\right]$, Δt denotes the sample period [26]. $w_{i}(k)$ is measurement noise and follows identically distributed Gaussian Distribution with variance $\sigma_{w}^{2}$.

Hence, the predicted state and prediction covariance can be expressed as:

(23) $${\hat{X}}_{i}(k|k-1)=F{\hat{X}}_{i}(k-1|k-1)$$

(24) $$P_{i}(k|k-1)=FP_{i}(k-1|k-1)F^{T}+\sigma_{LOS}^{2}{\mathrm{CC}}^{T}$$

The measurement residual is given by $E_{i}(k)=Z_{i}(k)-H{\hat{X}}_{i}(k|k-1)$, where $Z_{i}(k)$ indicates the measurement state of mobile node and H=[1,0].

And $S_{i}(k)$ is defined as:

(25) $$S_{i}(k)=HP_{i}(k|k-1)H^{T}+Q$$

where Q indicates the covariance matrix of measurement errors.

The Kalman gain is described as:

(26) $$K_{i}(k)=P_{i}(k|k-1)H^{T}{\left(S_{i}(k)\right)}^{-1}$$

The Kalman update equations can be expressed as:

(27) $${\hat{X}}_{i}(k|k)={\hat{X}}_{i}(k|k-1)+K_{i}(k)E_{i}(k)$$

(28) $${\hat{P}}_{i}(k|k)=P_{i}(k|k-1)-K_{i}(k)HP_{i}(k|k-1)$$

In the end, the algorithm pseudo code of KF can be expressed as Algorithm 4:

**Algorithm 4**. Description of KFInput: $X_{i}(k)\;Z_{i}(k)$
Output: ${\hat{X}}_{i}(k)\;{\hat{P}}_{i}(k)$
$\begin{array}{l} {\hat{X}}_{i}(k|k-1)=F{\hat{X}}_{i}(k-1|k-1)\\ P_{i}(k|k-1)=FP_{i}(k-1|k-1)F^{\prime }+\sigma_{LOS}^{2}CC^{\prime }\\ E_{i}(k)=Z_{i}(k)-H{\hat{X}}_{i}(k|k-1)\\ S_{i}(k)=HP_{i}(k|k-1)H^{T}+Q\\ K_{i}(k)=P_{i}(k|k-1)H^{T}{\left(S_{i}(k)\right)}^{-1}\\ {\hat{X}}_{i}(k|k)={\hat{X}}_{i}(k|k-1)+K_{i}(k)E_{i}(k)\\ {\hat{P}}_{i}(k|k)=P_{i}(k|k-1)-K_{i}(k)P_{i}(k|k-1)H \end{array}$


### 5.5. Unscented Kalman Filter

The main algorithm includes three procedures, namely, selection of sigma points, prediction and update [19]. To begin with, assume that the nonlinear system is:

(29) $$\{\begin{array}{c} X(k+1)=f(X_{i}^{j}(k),W_{k})\\ Z(k)=h(X_{i}^{j}(k),V_{k}) \end{array}$$

where $W_{k}$ and $V_{k}$ represents the Gaussian white noises in state variable X(k+1) and measurement variable Z(k).

The initial state vector can be denoted as $X_{i}$, which indicates the initial distances of the i-th beacon node [20], and ${\bar{x}}_{i}$ indicates the mathematical expectation of $X_{i}$:

(30) $${\bar{x}}_{i}=E(X_{i})$$

For the hard NLOS case, the state can be expressed as $X_{i}=[{\tilde{d}}_{i}^{h}(k),{\dot{\tilde{d}}}_{i}^{h}(k)]$; for the soft NLOS case, the state can be expressed as $X_{i}=[{\tilde{d}}_{i}^{s}(k),{\dot{\tilde{d}}}_{i}^{s}(k)]$, and the covariance of the i-th beacon node is defined as $P_{i}(k)$.

**Step 1**: Select sigma points. Assume that state vector is an n dimensional random variable, so the sample points (Sigma points) are 2n+1, and the estimated sigma points $X_{i}^{j}(k)$ can be described as:

(31) $$X_{i}(k-1)=\{\begin{array}{cc} {\bar{x}}_{i} & j=0\\ {\bar{x}}_{i}+{\left(\sqrt{\left(n+\lambda )P_{i}(k\right)}\right)}_{j} & j=1,\ldots ,n\\ {\bar{x}}_{i}-{\left(\sqrt{\left(n+\lambda )P_{i}(k\right)}\right)}_{j} & j=n+1,\ldots ,2n \end{array}$$

**Step 2**: Prediction. The system state equation is used to carry out the f(⋅) nonlinear transformation for each Sigma point, and the points set after transformation can be expressed as:

(32) $$X_{i}(k|k-1)=f(X_{i}(k-1),k)$$

where f(⋅) denotes bounded nonlinear state transfer function which is determined.

Then one-step prediction state value ${\hat{X}}_{i}(k-1)$ and covariance matrix $P_{i}(k)$ are given by:

(33) $${\hat{X}}_{i}(k|k-1)=\sum_{j=0}^{2n}\omega_{i}^{j}X_{i}^{j}(k|k-1)$$

(34) $$P_{i}(k|k-1)=\sum_{j=0}^{2N}\omega_{j}(X_{i}^{j}(k|k-1)-{\hat{X}}_{i}(k-1))\cdot {\left(X_{i}^{j}(k|k-1)-{\hat{X}}_{i}(k-1)\right)}^{T}+Q_{T}$$

(35) $$\{\begin{array}{cc} Q_{T}=\sigma_{LOS}^{2} & if\;{\tilde{d}}_{i}^{S}(k)\\ Q_{T}=\sigma_{NLOS}^{2} & if\;{\tilde{d}}_{i}^{H}(k) \end{array}$$

Weight coefficient ω can be given by:

(36) $$\omega_{j}=\{\begin{array}{cc} \frac{\eta }{N+\eta } & j=0\\ \frac{1}{2(N+\eta )} & j\neq 0 \end{array}$$

where η indicates turning parameters to control the spread of the sigma points.

A new Sigma points set can be produced according to a one-step prediction state value:

(37) $$X_{i}^{j}(k|k-1)=f(X_{i}^{j}(k-1))$$

Nonlinear transformation is performed to Sigma points set $X_{i}(k)$ by nonlinear observation equation:

(38) $$Z_{i}(k)=h(X_{i}(k),{\dot{d}}_{i}(k))$$

where h(⋅) denotes non-linear observation equation function.

The system’s predicted observation values $Z_{i}(k)$ are obtained by weighted sum:

(39) $$Z_{i}(k)=\sum_{j=0}^{2n}\omega_{i}\cdot Z_{j}(k)$$

**Step 3**: Update. The covariance is updated by:

(40) $$P_{i}^{x}(k)=\sum_{j=0}^{2n}\omega_{i}\cdot (X_{i}^{j}(k|k-1)-{\hat{x}}_{i}(k))\cdot {\left(X_{i}^{j}(k|k-1)-{\hat{x}}_{i}(k)\right)}^{T}$$

The covariance matrix of system measurement output variables is given by:

(41) $$P_{i}^{Z}(k)=\sum_{j=0}^{2n}\omega_{i}\cdot (Z_{i}^{j}(k|k-1)-{\hat{Z}}_{i}(k))\cdot {\left(Z_{i}^{j}(k|k-1)-{\hat{Z}}_{i}(k)\right)}^{T}+R$$

where R denotes the covariance of the observation noise.

And the Kalman gain $K_{i}(k)$ is expressed as:

(42) $$K_{i}(k)=P_{i}^{x}(k)P_{i}^{z}{\left(k\right)}^{-1}$$

The updated state ${\hat{X}}_{i}(k)$ and a posteriori covariance $P_{i}(k)$ are expressed as:

(43) $${\hat{X}}_{i}(k)={\hat{X}}_{i}(k|k-1)+K_{i}(k)\cdot (Z_{i}(k)-{\hat{Z}}_{i}(k))$$

(44) $$P_{i}(k)=P_{i}(k|k-1)-K_{i}(k)P_{i}^{Z}(k)K_{i}^{T}(k)$$

The final NLOS measurements after filtering can be expressed as:

(45) $${\hat{d}}_{i}^{NLOS}(k)=\left({\hat{d}}_{i}^{h}(k)+{\hat{d}}_{i}^{s}(k)\right)/2$$

where ${\hat{d}}_{i}^{h}(k)$ and ${\hat{d}}_{i}^{s}(k)$ denote hard NLOS measurements and soft ones after UKF filtering.

### 5.6. Combination and Location Estimation

Two sorts of estimated distances from KF and UKF can be obtained, respectively, and the final estimated distance ${\hat{d}}_{i}(k)$ is updated as follows:

(46) $${\hat{d}}_{i}(k)=\{\begin{array}{cc} {\hat{d}}_{i}^{LOS}(k) & if\;LOS\\ {\hat{d}}_{i}^{NLOS}(k) & if\;NLOS \end{array}$$

A maximum likelihood localization method is utilized to estimate the mobile position according to the distance after filtering. The coordinate of the i-th beacon node can be expressed as $P_{i}=(x_{i},y_{i})\;0<i\leq N$. 

At time k, the position of a mobile node is $\hat{P}(k)=(\hat{x}(k),\hat{y}(k))$. The relation between coordinates of the mobile node and the coordinate of the i-th of beacon node can be modeled as [27]:

(47) $$\{\begin{array}{c} {\left(x_{1}-\hat{x}(k)\right)}^{2}+{\left(y_{1}-\hat{y}(k)\right)}^{2}={\left({\hat{d}}_{i}(k)\right)}^{2}\\ \vdots \\ {\left(x_{N}-\hat{x}(k)\right)}^{2}+{\left(y_{N}-\hat{y}(k)\right)}^{2}={\left({\hat{d}}_{N}(k)\right)}^{2} \end{array}$$

The equation above can be represented by linear equation AP=B, where A and B are expressed as follows:

(48) $$A=\left[\begin{array}{c} \left(x_{1}-x_{2})(y_{1}-y_{2}\right)\\ \left(x_{1}-x_{3})(y_{1}-y_{3}\right)\\ \vdots \\ \left(x_{1}-x_{N})(y_{1}-y_{N}\right) \end{array}\right]\;B=\left[\begin{array}{c} {\hat{d}}_{2}{\left(k\right)}^{2}-{\hat{d}}_{1}{\left(k\right)}^{2}-(x_{2}^{2}+y_{2}^{2})+(x_{1}^{2}+y_{1}^{2})\\ {\hat{d}}_{3}{\left(k\right)}^{2}-{\hat{d}}_{1}{\left(k\right)}^{2}-(x_{3}^{2}+y_{3}^{2})+(x_{1}^{2}+y_{1}^{2})\\ \vdots \\ {\hat{d}}_{N}{\left(k\right)}^{2}-{\hat{d}}_{1}{\left(k\right)}^{2}-(x_{N}^{2}+y_{N}^{2})+(x_{1}^{2}+y_{1}^{2}) \end{array}\right]$$

Finally, the simplified estimated coordinate $\hat{P}(k)$ of a mobile node at time k can be derived as:

(49) $$\hat{P}(k)={\left[\hat{x}(k),\hat{y}(k)\right]}^{T}={\left(A^{T}A\right)}^{-1}A^{T}B$$

## 6. Simulation and Experiment Results

### 6.1. Simulation Results

The purpose of simulation is to simulate a complicated environment by using different distributions and to conduct numerous experiments to obtain statistical results. The simulation results will be evaluated and shown in this section. Conventional IMM (without data fusion) [3], KF and UKF are compared with our algorithm. The simulation platform is MATLAB. Positions of beacon nodes applied with Monte Carlo in each run are randomly distributed in a square of 100 m × 100 m [25]. Propagation of NLOS errors between beacon nodes and the mobile node is randomly generated with probability φ, and every simulation is produced over 1000 times Monte Carlo runs. Root Mean Square Error (RMSE) is chosen to evaluate the performance of our algorithm, and RMSE is modeled as:

(50) $$RMSE=\sqrt{\frac{1}{T_{n}K}\sum_{i=1}^{T_{n}}\sum_{k=1}^{K}\left({\left({\hat{x}}_{i}(k)-x_{i}(k)\right)}^{2}+{\left({\hat{y}}_{i}(k)+y_{i}(k)\right)}^{2}\right)}$$

where $T_{n}$ indicates the times of Monte Carlo runs and K denotes the observation number of mobile node, and here, we assume $T_{n}=1000,\;K=100$.

#### 6.1.1. The NLOS Errors Obey a Gaussian Distribution

Table 2 represents the default parameters of NLOS errors obeying a Gaussian Distribution in the simulation.

**Figure 3:**
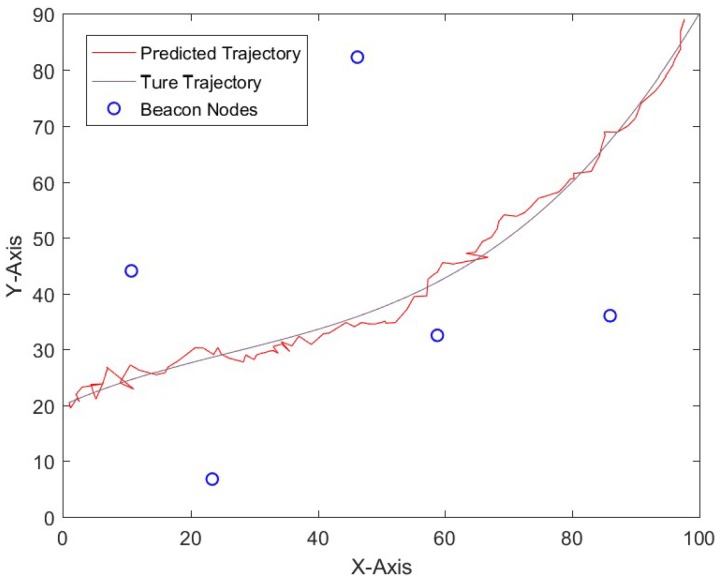
The localization result of TF-FCM.

Figure 3 shows the localization results of a one-time run of TF-FCM, and that the number of beacon nodes is 5. It can be seen from the figure that TF-FCM achieves a fulfilling result even if the beacon nodes are randomly distributed in this area.

**Figure 4:**
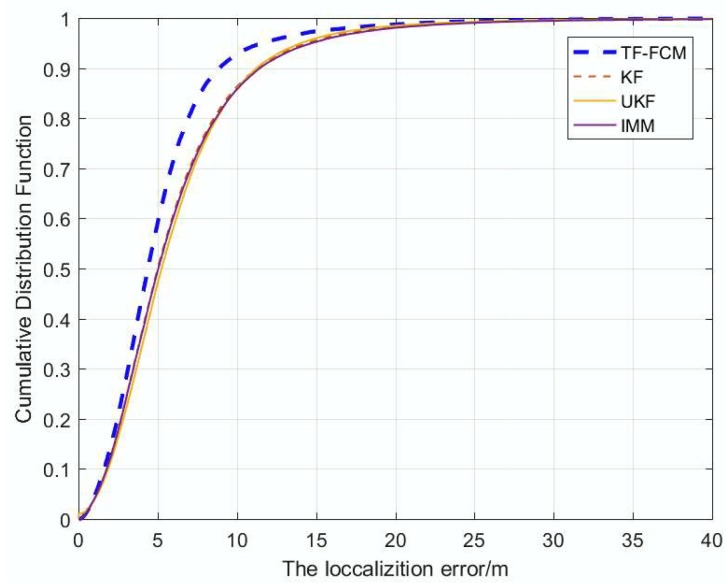
The localization error versus CDF.

Figure 4 displays the cumulative distribution function (CDF) of the localization error under the condition of 1000 times Monte Carlo runs, and the number of beacon nodes is five; from the figure, it can be seen that ninety percent of TF-FCM errors are less than 8.8 m; by contract, the errors of UKF, IMM and KF are 11.1 m, 11.2 m and 11.4 m, respectively. Most of the localization error of TF-FCM is much lower than UKF, IMM and KF, which indicates the high localization accuracy of TF-FCM.

**Figure 5:**
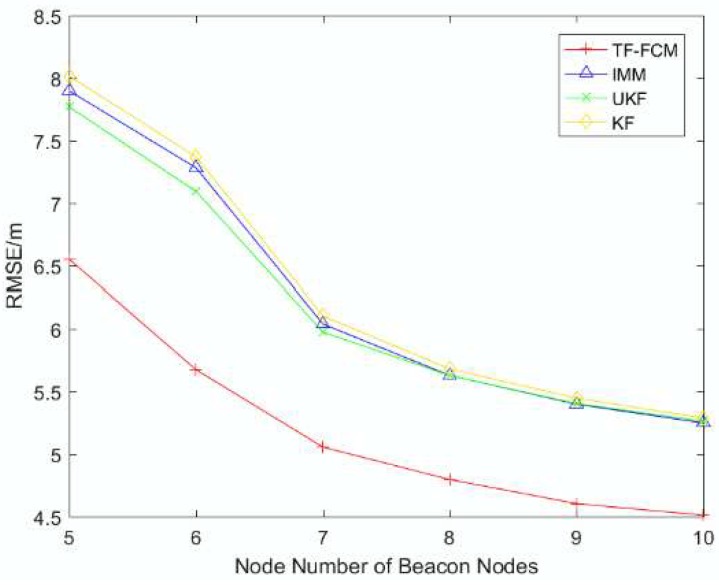
The RMSE versus the number of beacon nodes.

To better describe the robustness and localization accuracy of TF-FCM as the number of beacon nodes changes, Figure 5 shows RMSE results with the number of beacon nodes varying from 5 to 10. It is obvious that TF-FCM has a better performance than KF, UKF and IMM with increasing numbers of beacon nodes. If the number of beacon nodes is five, the improvement of TF-FCM location accuracy compared with KF, UKF and IMM is 17.7%, 16.0% and 16.8%, respectively. In the figure, we can see that the KF and IMM have the same performance, and UKF performs better when the number of beacon nodes is low. However, when the number of beacon nodes is high, KF, IMM and UKF almost have the same localization accuracy.

**Figure 6:**
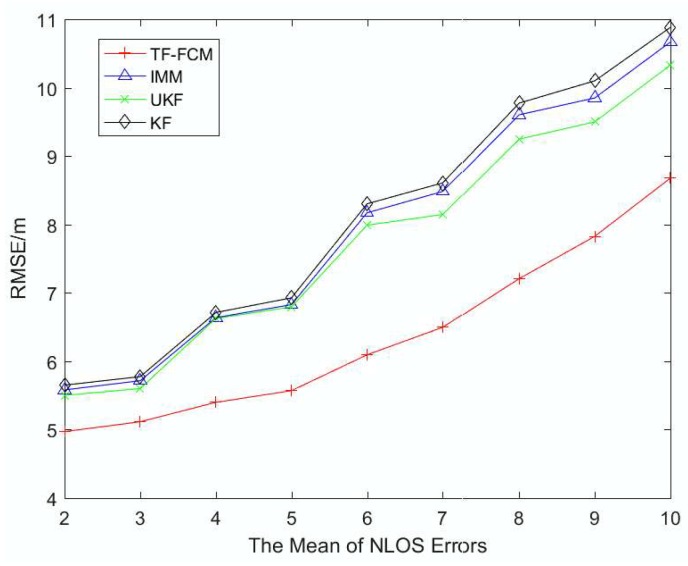
The RMSE versus mean of NLOS errors.

**Figure 7:**
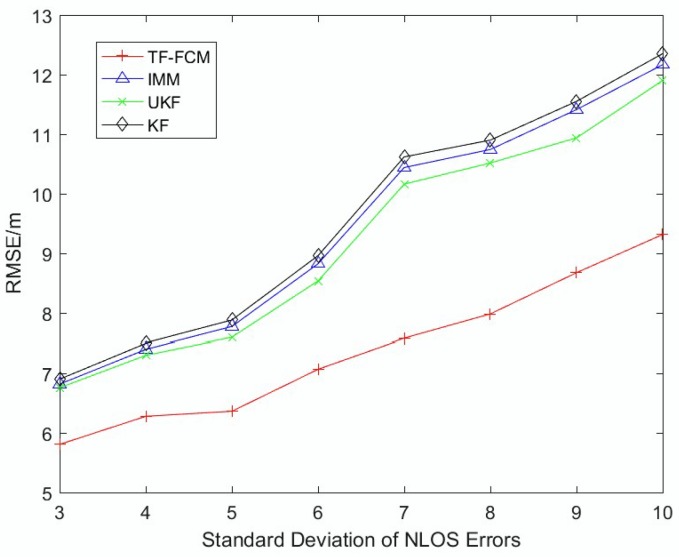
The RMSE versus standard deviation of NLOS errors.

To illustrate the robustness of TF-FCM as the NLOS errors get larger, Figure 6 and Figure 7 display the RMSE result with mean of NLOS errors varying from 2 to 10 and the standard deviation adapting from 3 to 10. It can be seen from the figure that the trend is on the rise with the increase of NLOS errors. The average RMSE values of TF-FCM, UKF, IMM and KF are 7.16 m, 8.78 m, 8.96 m and 9.10 m, respectively, and when the standard deviation of NLOS errors gets larger (3–10), the accuracy rate of TF-FCM improvement compared with IMM, UKF, KF is 19.9%, 18.3% and 21.2%, respectively. In Figure 6 and Figure 7, we can see that as NLOS errors get larger, the RMSE of TF-FCM still stays much lower than other algorithms, which indicates the strong robustness of our algorithm.

**Figure 8:**
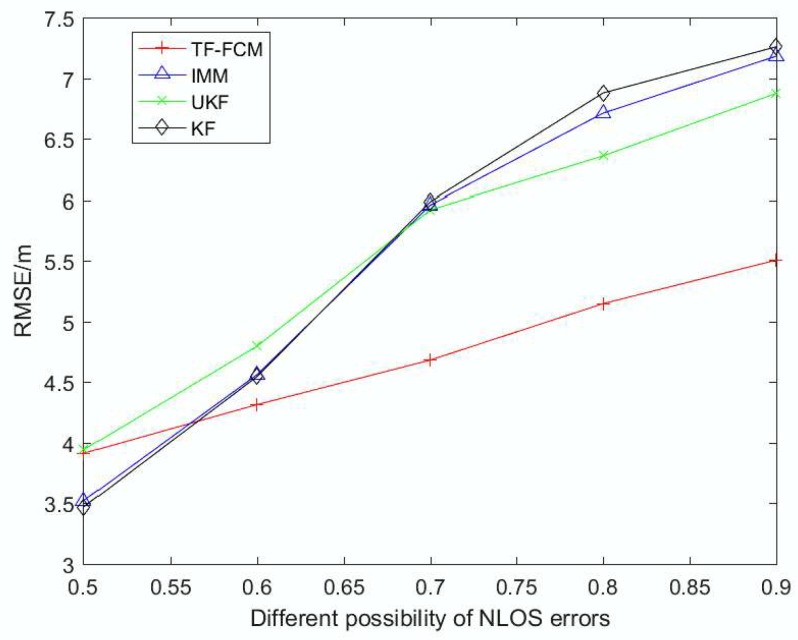
The RMSE versus the probability of NLOS errors.

To clearly denote the robustness as the probability of NLOS errors get larger, Figure 8 illustrates the RMSE result when the NLOS probability varies from 0.5 to 0.9. It can be clearly seen that when the NLOS probability is small, the IMM and KF is slightly better than UKF and TF-FCM, but with the probability of NLOS getting larger, the effect of TF-FCM is much better than KF and IMM. The result demonstrates that our algorithm is much better and has strong robustness when coping with the situation that contains many NLOS errors.

#### 6.1.2. The NLOS Errors Obey Uniform Distribution

The default parameters of NLOS errors obeying Uniform Distribution are shown as Table 3.

**Figure 9:**
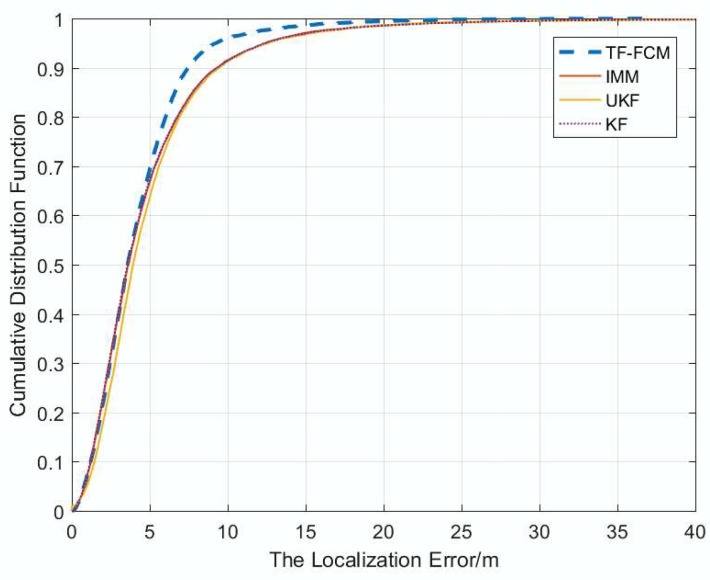
The localization error versus CDF.

Figure 9 shows the cumulative distribution function of localization at 1000 times under Uniform Distribution obeyed U(3,8). When localization errors are small, four filters almost have the same filtering effect, but once the localization errors get larger, the performance of TF-FCM is better than the others, and it can be seen that the ninety percent of TF-FCM errors is less than 7.5 m; by contrast, the errors of UKF, IMM and KF are 9.8 m, 9.7 m and 9.7 m, respectively.

**Figure 10:**
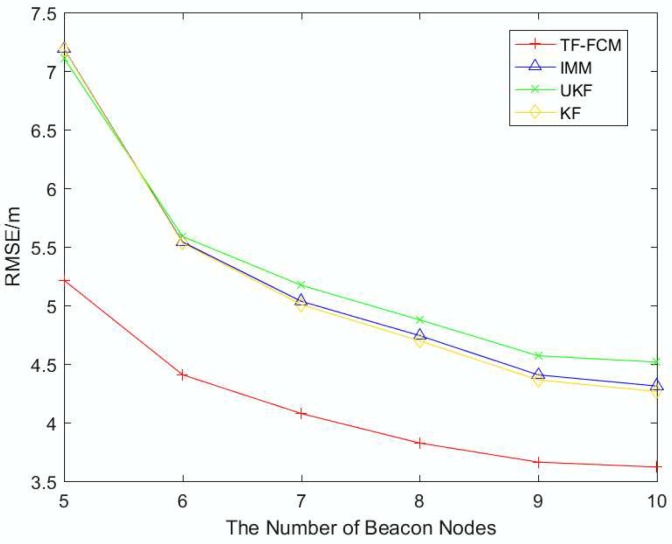
The RMSE versus the number of beacon nodes.

To better describe the robustness and localization accuracy of TF-FCM as the number of beacon nodes changes, Figure 10 shows the RMSE result with the beacon nodes varying from 5 to 10 under Uniform Distribution. It can be seen from the result that the four methods show a downward trend with the increase of the number of nodes, and the performance of TF-FCM is the best compared with other methods. The average RMSE errors of TF-FCM, UKF, IMM, KF are 4.13 m, 5.20 m, 5.31 m and 5.17 m, respectively.

**Figure 11:**
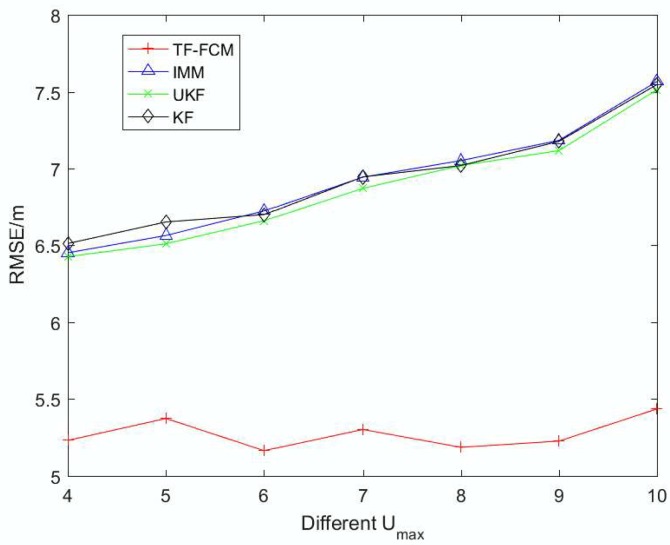
The RMSE versus $U_{\max}$ of NLOS errors.

To illustrate the robustness of TF-FCM as the NLOS errors get larger, Figure 11 shows the RMSE result with NLOS parameter $U_{\max}$ varying from 4 to 10. It can be senn from the result that the trend of IMM, KF and UKF is on the rise as $U_{\max}$ becomes larger. While the tendency of TF-FCM remains steady, it indicates the strong robustness of our algorithm. The average RMSE values of TF-FCM, UKF, IMM, KF are 5.27 m, 6.99 m, 7.04 m and 7.05 m, respectively.

#### 6.1.3. The NLOS Errors Obey Exponential Distribution

The default parameters obeying Exponential Distribution are displayed as Table 4.

**Figure 12:**
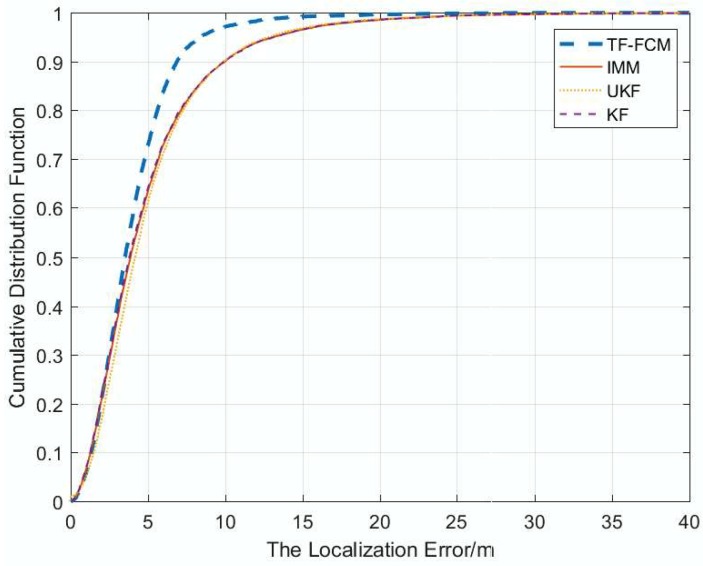
The localization error versus CDF.

Figure 12 shows that the cumulative distribution function of localization at 1000 times under Exponential Distribution when the number of beacon nodes is five. The result shows that ninety percent of TF-FCM errors are less than 6.1 m, while the errors of UKF, IMM, KF are 7.2 m, 11 m and 11.1 m, respectively. Most of the localization error of TF-FCM is much lower than UKF, IMM and KF, which indicates the high localization accuracy of TF-FCM.

**Figure 13:**
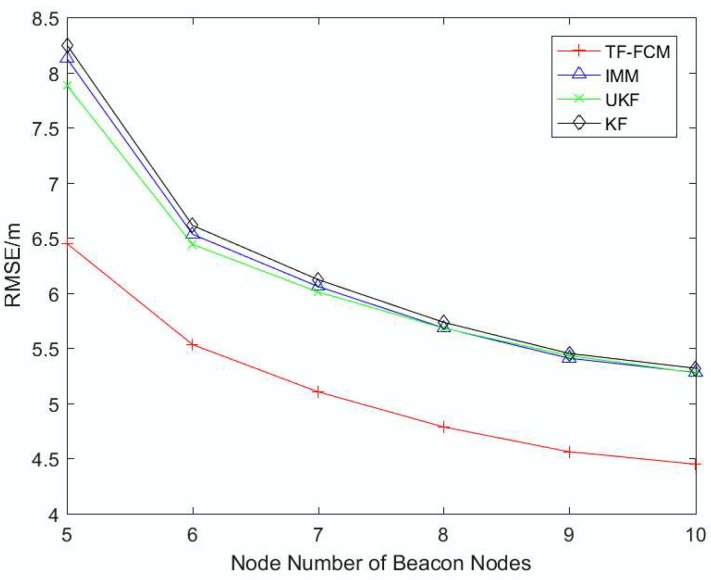
The RMSE versus the number of beacon nodes.

To illustrate the robustness of TF-FCM as the NLOS errors get larger, Figure 13 shows the RMSE result with the number of beacon nodes varying from 5 to 10. It can be clearly seen that the RMSE of TF-FCM is the smallest with the increase of the beacon node number. The overall trend declines as the number of beacon nodes increases, while the drop of TF-FCM is much slower than other algorithms, and the average RMSE of TF-FCM, UKF, IMM, KF is 3.94 m, 5.20 m, 5.23 m, and 5.31 m.

### 6.2. Experiment Results

#### 6.2.1. Localization Results Analysis

**Figure 14:**
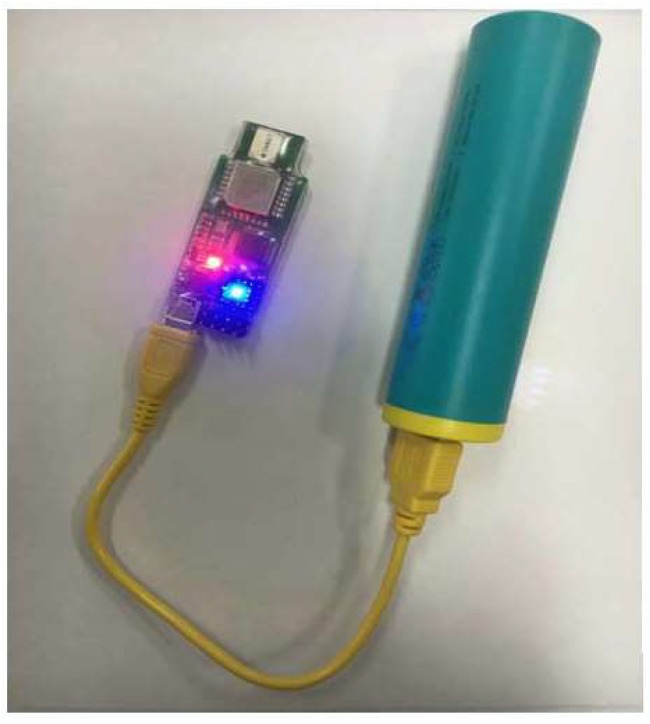
The UWB node.

In order to test the performance of TF-FCM in actual scenarios, an experiment is conducted under indoor conditions. The signal transmission between beacon nodes and mobile nodes is based on the carrier-less communication technology Ultra-Wideband (UWB) which uses a non-sinusoidal narrow pulse to transfer data. In recent years, UWB has been widely applied in close-range accurate indoor positioning. The principle of UWB ranging is two-way time-of-flight (TW-TOF) using a timestamp to approximate the distance between nodes, and the UWB device used in this paper is shown in Figure 14. To the left of the figure is the UWB node and to the right of the figure is the power supply of the node.

**Figure 15:**
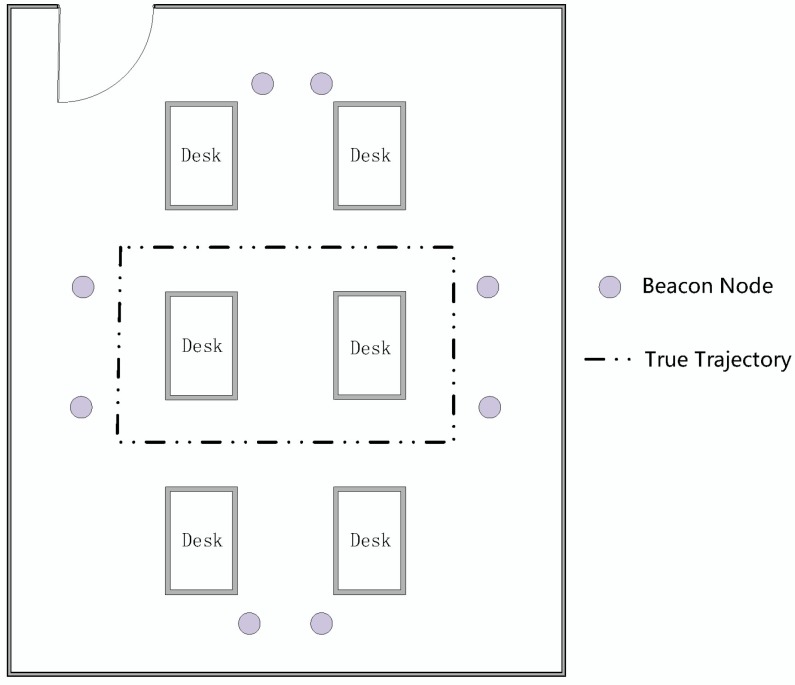
The floor plan of the indoor environment.

In order to avoid ground reflection, the beacon nodes and the mobile node are placed 1.2 m above the ground. As shown in Figure 15, eight beacon nodes are placed around the desks in a 5 m × 8 m room. The height of each desk is 1.3 m. The mobile node moves following the trajectory at a constant velocity. Every 40 cm, one sampling site is recorded and there are 31 sampling sites in our experiment shown as dotted lines in the figure. Twenty measurements are collected at the sampling site at one time, and the average distance of each sampling site is utilized by the localization algorithm.

**Figure 16:**
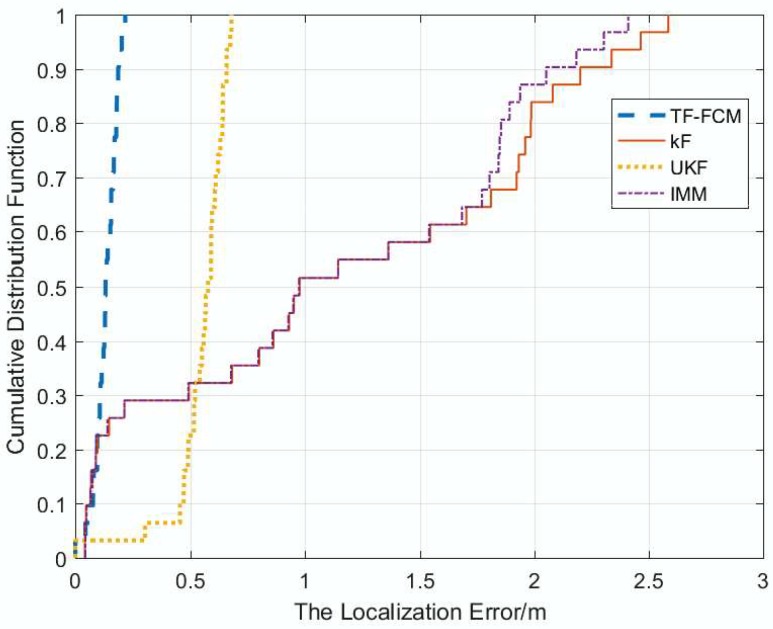
The localization errors versus CDF.

The cumulative function of localization error under experimental conditions is displayed as Figure 16. From the figure, it is obvious that TF-FCM has the highest accuracy in comparison with other methods. The localization error of KF and IMM is more than 2 m; in contrast, the error of UKF is less than 1 m; especially, the error of TF-FCM is less than 0.5 m. The error over 90 percent of TF-FCM, UKF, IMM, KF is 0.23 m, 0.65 m, 2.43 m, 2.56 m, respectively. To better describe the localization accuracy of four algorithms, the comparison table of average localization error is shown as Table 5. From the experiment, it can be clearly seen that there is high precision and the strong robustness of TF-FCM.

In the simulation experiment, when the NLOS errors obey Gaussian, Uniform and Exponential Distributions, respectively, according to Figure 4, Figure 9 and Figure 12, the proposed algorithm has the highest positioning accuracy compared with other methods. In the actual experiment, the localization error of TF-FCM is the lowest compared with other methods according to Figure 16.

#### 6.2.2. Processing Time Comparison

Table 6 shows the running time of TF-FCM, UKF, KF and IMM. The four algorithms are performed using Matlab 2016a and tested on a Windows 10 home edition with AMD A10-8700P Randeon R6, 10 Compute Cores 4C + 6G 1.80 GHz 8.00 GB RAM.

## 7. Conclusions

In this paper, an algorithm based on triple-filter mixed KF and UKF, using residual analysis, voting and FCM is proposed, which is utilized to mitigate NLOS errors and enhance the accuracy of localization in mixed LOS and NLOS environments. Residual analysis is employed to identify if the propagation condition is NLOS or not. For LOS case, KF is adopted to process. For NLOS case, firstly NLOS correction algorithm based on voting is used to restrict the NLOS errors and correct the measurements. Then, the NLOS error classification method based on FCM is proposed to classify the NLOS errors into two categories: hard NLOS measurements and soft ones. The simulation result shows great advantage when processing the situation with complicated NLOS errors, including Gaussian distribution, exponential distribution and uniform distribution compared with IMM, UKF and KF. The performance of TF-FCM has significantly improved in accuracy and robustness when dealing with rough wireless environments. Furthermore, TF-FCM could efficiently mitigate the NLOS effects compared with other classical methods. An experiment is conducted to verify our algorithm, and the result shows the high accuracy and strong robustness of TF-FCM. 

## Figures and Tables

**Table 1:** List of key notations.

Symbol	Explanation	Symbol	Explanation
N	the number of beacon nodes	$N_{C}$	the combination of beacon nodes
$S_{k}$	the index of every combination	${\hat{X}}_{k}$	the estimated position of every combination
Res(X^k,Sk)	the residual of $S_{k}$	Res¯(X^k,Sk)	the average residual of $S_{k}$
$d_{i}(k)$	the true distance between mobile node and i-th beacon node at time k	${\hat{d}}_{i}(k)$	the estimated distance between mobile node and the i-th beacon node at time k
$\hat{P}(k)$	the final position of mobile node at the time k	${\tilde{d}}_{i}(k)$	the corrected range after voting
$n_{LOS}$	the LOS errors	$n_{NLOS}$	the NLOS errors
C(m,n)	the location of each voting grid	$C^{*}$	the estimated location of mobile node
V(m,n)	the voting matrix	$b_{i}(m,n)$	the number of votes increased at C(m,n)
v	the number of the estimated location of mobile node	${\bar{C}}^{*}$	the average of the mobile nodes
M	the number of distances in a time slot	c	the number of cluster centers
v	the cluster centers set	$\mu_{ij}$	the membership between i-th distance ${\hat{d}}_{i}(k)$ at time k and the j-th cluster center
$v_{i}$	the i-th cluster center	$d_{ij}$	the Euclidean distance between ${\hat{d}}^{j}(k)$ and $v_{i}$
$X_{i}(k)$	the state of the i-th beacon node at time k	$P_{i}(k)$	the covariance of the i-th beacon node at time k
$K_{i}(k)$	Kalman gain of the i-th beacon node at time k	$Z_{i}(k)$	the measured state of the i-th beacon node at time k
$\omega_{i}$	weight efficient of the i-th sigma point	${\hat{s}}_{i}(k)$	the state of the i-th beacon node at time k

**Table 2:** The default parameters of a Gaussian Distribution.

Parameter	Symbol	Default Values
The number of beacon nodes	N	5
The probability of NLOS propagation	φ	0.7
The standard deviation of measurement noise	$\sigma_{LOS}$	1
The NLOS errors	$N(\mu_{NLOS},\sigma_{NLOS}^{2})$	$N(3,5^{2})$
The number of sample points	K	100
The number of Monte Carlo runs	$T_{n}$	1000

**Table 3:** The default parameters of Uniform Distribution.

Parameter	Symbol	Default Values
The number of beacon nodes	N	5
The probability of NLOS propagation	φ	0.7
The standard deviation of measurement noise	$\sigma_{LOS}$	1
The NLOS errors	$U(U_{\min},U_{\max})$	U(3,8)
The number of sample points	K	100
The number of Monte Carlo runs	$T_{n}$	1000

**Table 4:** The default parameters of Exponential Distribution.

Parameter	Symbol	Default Values
The number of beacon nodes	N	5
The probability of NLOS propagation	φ	0.7
The standard deviation of measurement noise	$\sigma_{LOS}$	1
The NLOS errors	$\mu_{\mathrm{NLOS}}$	5
The number of sample points	K	100
The number of Monte Carlo runs	$T_{n}$	1000

**Table 5:** Comparison table of average localization error.

Algorithm	Average Localization Error/m
TF-FCM	0.192
UKF	0.555
IMM	1.366
KF	1.434

**Table 6:** Running time of each algorithm.

Algorithm	Running Time/m
TF-FCM	0.192
UKF	0.555
IMM	1.366
KF	1.434
